# A Method for Determination of Transport Efficiency in Laser Ablation Inductively Coupled Plasma Mass Spectrometry for Tissue Analysis

**DOI:** 10.1021/acs.analchem.5c01306

**Published:** 2025-06-15

**Authors:** Jaromír Stráník, Vilém Svojanovský, Julie Weisová, Kateřina Uhrová, David Clases, Antonín Hlaváček, Jan Preisler

**Affiliations:** † Department of Chemistry, Faculty of Science, 37748 Masaryk University, 625 00 Brno, Czech Republic; ‡ Institute of Analytical Chemistry of the Czech Academy of Sciences, 602 00 Brno, Czech Republic; § NanoMicroLab, 27267 University of Graz, 8010 Graz, Austria

## Abstract

Recent studies have demonstrated the applicability of the “single particle” mode in laser ablation–inductively coupled plasma–mass spectrometry to map and size particles simultaneously. The transport efficiency (TE) is an important parameter in this configuration and affects the detection of individual nanoparticles, reliability of nanoparticle characterization, and related applications. This study introduces a novel method for the precise determination of TE, based on counting upconversion nanoparticles from gels characterized by fluorescent microscopy. The method was found to be most suitable for the 2940-nm laser ablation system, achieving virtually quantitative nanoparticle desorption, with TE primarily governed by ablation cell design and aerosol transport efficiency. With the 213-nm laser, attention had to be paid to incomplete desorption and possible nanoparticle redeposition at low laser fluences to avoid variability in TE measurements. Finally, use of the 193-nm laser-induced nanoparticle disintegration, resulting in elevated baseline noise and lower sensitivity, which prevented the use of this approach for the determination of TE. This study highlights the versatility of the proposed method, while also identifying its limitations, in terms of wavelength and fluence.

## Graphical abstract

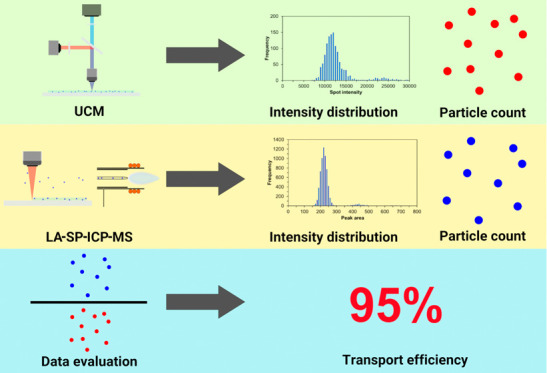

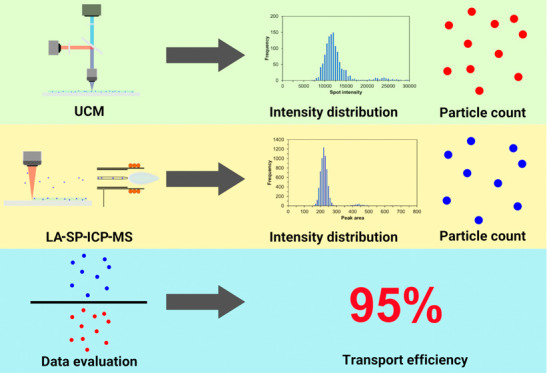

## Introduction

Inductively coupled plasma–mass spectrometry (ICP-MS) stands at the forefront of analytical techniques, pivotal in advancing our understanding of elemental composition across diverse fields ranging from geological applications to environmental monitoring, as well as biological research. Recognized for its excellent sensitivity and precision, ICP-MS enables the quantification of trace elements with remarkable accuracy and, now more often, the characterization of nanoparticles and microparticles. In this analytical landscape, the measurement of transport efficiency (TE) is crucial. TE, which is defined as the ratio of analyte transported from the matrix to the analyte fraction reaching the detector, is a fundamental parameter for the concentration sensitivity of the ICP-MS experiment.

Various methods have been developed to determine TE, particularly for nebulizer sample introduction systems.^1^ In single-particle (SP) nebulizer measurements, TE is essential for accurately determining particle number and size.^2,3^ Therefore, with the growth of SP analysis by ICP-MS, techniques analyzing particle frequency or particle size of stable (metallic) nanoparticles have become increasingly popular for TE determination.^2–4^ Additionally, dynamic mass flow method was proposed in 2020, which is based on measuring directly and continuously the weight of sample uptake and the weight of sample reaching the plasma online over time while the ICP-MS system is in equilibrium and was used for the characterization of nanoparticles.^5^

Determining TE for laser ablation–inductively coupled plasma–mass spectrometry (LA-ICP-MS) is less common and more challenging. Aerosol generation and transport in LA-ICP-MS is a complex process that can be influenced by sample morphology, sample composition, laser parameters, carrier gas, particle size and shape, and other factors related to sample atomization and mass spectrometer setup.^6–9^

Existing methods adapted from solution analysis are not directly applicable. Standard techniques involve capturing aerosols using filters or multilevel impactors, which are often accompanied by an optical particle counter.^10^ This is typically followed by sample weighing before and after the ablation process and characterizing the captured aerosol.^10–15^ With the growing interest in ablating intact nanoparticles in the SP mode,^16–20^ the particle size method has recently found application in laser ablation (LA) systems.^20^ Additionally, TE has been determined by applying a suspension of nanoparticles of a known number concentration to tissue sections.^21^ In our earlier work, we presented a method for TE determination in which we precisely deposited gold nanoparticles using a piezoelectric dispenser on gelatin sections for LA-SP-ICP-MS analysis and silicon wafers for reference scanning electron microscopy.^19^

However, conventional methods for TE determination have limitations. They often require special equipment and describe the aerosol fate only in the sample introduction system rather than the whole ICP-MS system. Some rely on particle characteristics provided by manufacturers, which are often only approximated, primarily due to an unknown degree of aggregation. Challenges such as nanoparticle suspension aging, pipetting errors, and nanoparticle adsorption during sample preparation underscore the need for a reliable reference method, mainly when precision and accuracy are paramount in TE determination. Recently, nanoparticles embedded in thin polymer films were applied for their characterization and calibration of LA-SP-ICP-MS.^22^

We present a method for determining transport efficiency in LA-ICP-MS using agarose layers containing photon-upconversion nanoparticles (NPs). These lanthanide-doped nanocrystals absorb infrared light (976-nm) and emit at shorter near-infrared and visible wavelengths in a nonlinear photoluminescence process known as photon-upconversion. This unique property enables background-free imaging and accurate single-nanoparticle counting via photon-upconversion microscopy, serving as a reference method. The method is compatible with laser wavelengths (2940, 213, and partially 193-nm), providing robust TE evaluation in LA-SP-ICP-MS systems.

## Experimental Section

The detailed information on NP preparation, sample agarose layer preparation, and upconversion microscopy (UCM) is available in the Supporting Information and has been reported previously.^23^

### 2940-nm Laser Ablation System

A laboratory-built laser ablation system equipped with an OPO emitting 2940-nm was utilized. The laser spot was characterized by a top-hat-like profile, a diameter of 30 μm, and a maximum fluence of 42 J/cm^2^. Using infrared radiation, the agarose layer can be fully ablated without particle disintegration over a wide fluence range. We chose the highest energy level to completely ablate the agarose layer and leave no NPs on the slide. For TE assessment, 1 mm × 1 mm rasters were employed. Unless stated otherwise, these rasters were scanned using a flyback mode (bidirectional scanning pattern where the laser ablates in one direction, then moves rapidly back without firing before starting the next line scan) at a rate of 150 μm/s, line spacing of 15 μm (i.e., 50% overlap of adjacent lines), and a laser repetition rate of 20 Hz. A mechanical shutter was employed to block the laser beam between the lines. A rectangular ablation cell, sized at 4.7 mm × 76.1 mm × 3.0 mm (width × length × height), was purged with He as the carrier gas at a rate of 1.6 L/min. More-detailed information about the laser ablation system is available in our earlier publication.^19^

For LA-SP-ICP-MS imaging (MSI), the conditions were slightly adjusted. The repetition rate of the laser was reduced from 20 Hz to 10 Hz, leading to 50% overlap between consecutive laser spots, resulting in a pixel size of 15 μm × 15 μm.

### 213-nm Laser Ablation System

The glass slides with the agarose layers were inserted into a 213-nm laser ablation system LSX-213 G2+ (Teledyne Photon Machines, Inc., Omaha, NE) equipped with a 213-nm Q-switched Nd: YAG laser with a maximal pulse energy of >5 mJ, a pulse width of <5 ns, a repetition rate of 20 Hz, equipped by HelEx II Active 2-Volume Cell. Areas of 1 × 1 mm^2^ were scanned in flyback mode with a scan speed of 30 μm/s, a spot size of 100 μm, an overlap between adjacent lines of 50%, a fluence of 0.2 J/cm^2^, a He gas flow cell of 0.6 L/min, and a He gas flow funnel of 0.3 L/min. We used the same MS setup as in the 2940-nm LA ICP MS, with minor changes to the tubing and Ar flow rate, detailed in the Supporting Information.

### 193-nm Laser Ablation System

The 193-nm excimer laser ablation system Analyte G2 (Teledyne Photon Machines, Omaha, NE, USA) was coupled to either quadrupole or the time-of-flight (TOF) ICP mass spectrometer. Both systems operated under identical ablation conditions, with desorption achieved by scanning circular laser spots of 50 μm diameter using a laser fluence of 0.2 J/cm^2^, a repetition rate of 100 Hz, and a scanning speed of 150 μm/s. The ablation cell was operated with a helium flow rate of 0.4 L/min, supplemented by an additional 0.2 L/min helium flow through the funnel inlet.

### ICP-MS Data Evaluation

Detailed information on the quadrupole and TOF ICP-MS setups, data acquisition and processing, including correction of coincidence of multiple NPs is available in the Supporting Information. The TE was calculated based on the ratio of the number of NPs detected by UCM and LA-SP-ICP-MS, according to eq 1:

1 $$\eta \;\mathrm{(\%)}=\frac{X_{\mathrm{ICP‐MS}}}{X_{\mathrm{UCM}}}\times 100$$

where η is the transport efficiency, *X*_ICP‑MS_ is the average number of NPs obtained from the ICP-MS, and *X*_UCM_ is the average number of NPs obtained from the analysis, with the UCM data being considered the reference value.

## Results and Discussion

Two approaches were examined to determine transport efficiency. In the “Random area” approach, square areas imaged by UCM and LA-SP-ICP-MS were chosen randomly on the same glass slide with the agarose layer. In the “Grid area” approach, an identical square area defined by the grid generated in the agarose layer was inspected by UCM and LA-SP-ICP-MS. Three lasers, with wavelengths of 2940, 213, and 193-nm, were used for ablation. Applicability of agarose as a standard for TE determination of soft tissues was confirmed by comparing numbers of NPs desorbed from agarose and mouse brain tissue sections. Details are available in the Supporting Information (Figure S2).

### Random Area Approach

As mentioned above, TE was calculated from NP counts determined by UCM and LA-SP-ICP-MS in randomly chosen square areas in this approach. First, the agarose layer was characterized using UCM, by measuring 10 random square areas with an edge length corresponding to the maximum field of view of the microscope (0.667 mm). NP counts were recalculated to a 1 mm^2^ area with the same particle density for TE determination. The average number of NPs was 7000 ± 300 (average ± SD) per 1 mm^2^ area. The RSD of 3.9% indicates a fairly random dispersion in the NP distribution, being higher than the expected RSD (1.2%) for perfectly random dispersed particles, calculated from Poisson distribution (*N* ^1/2^/*N*, where *N* is the number of NPs).^24^

#### 2940-nm LA-ICP-MS

During IR ablation, no NP disintegration was observed, even at the maximum laser fluence of the tested range (7.1–87 J/cm^2^); disintegration would have manifested as an increase in the baseline around the peak, a fragmentation (large SP signal widths), and/or a shift of the histogram mode to lower values. Apparently, the laser energy was absorbed primarily by agarose, and intact NPs were softly desorbed. The signal transients are available in the Supporting Information (Figures S4A and S4B). NPs generated typical peaks with a duration of 200–500 μs. Based on the detected signals, a histogram of NP peak areas was constructed, and the number of NPs in the ablated area of an agarose layer was determined.

For the TE determination, a 1 mm × 1 mm agarose layer area was ablated by the 2940-nm laser ablation system. Ablation was performed from 3 different areas. The average NP counts and calculated TE are shown in Table 1. For 2940-nm LA, RSDs calculated from three repetitions (0.8%) are comparable to the error in determining the number of NPs using the Poisson distribution (1.2%). Given that TE for the 2940-nm laser is nearly quantitative, assuming 100% TE introduces only a minor error in NP quantification and characterization of NP. In a recent study, transport efficiencies up to 95% were also reported for LA-SP-ICP-MS analysis of microplastics.^25^ These comparable efficiencies were obtained under different experimental conditions, including distinct ablation cell geometries, carrier gas flow rates, and transport line configurations. In addition, the particles were significantly larger (2–4.5 μm microplastics) compared to the ∼70–80-nm UCNPs analyzed in our study. These differences highlight that while high TE is achievable across diverse setups and particle types, careful system-specific optimization remains crucial for maximizing NP transport efficiency.

**Table 1:** Comparison of Detected NP Numbers and Transport Efficiencies for 2940- and 213-nm Lasers Using Random Area and Grid Approaches, with and without Reablation

laser wavelength (nm)	average number of NPs ± SD	average number of NPs from reablation ± SD	TE (%)	TE + reablation (%)
**Random Area Approach**				
2940	6600 ± 100	60 ± 10	94 ± 4	95 ± 4
213	5600 ± 200	310 ± 60	79 ± 6	84 ± 6
**Grid Area Approach**				
2940	1270 ± 10	–	94 ± 1	–
213	1080 ± 20	130 ± 70	74 ± 1	83 ± 4

#### 213-nm LA-ICP-MS

To demonstrate wider applicability, the TE of the commercial 213-nm LA system was determined. Ablation of intact NPs with the 213-nm system is more challenging than observed with the 2940-nm system. The absorption of NPs at 213-nm is presumably stronger; NPs can absorb energy and disintegrate. Hence, the window of laser fluence at which intact particles are released, but not ablated, is narrower. Therefore, the ablation conditions had to be carefully adjusted. The laser fluence was set to 0.2 J/cm^2^ to minimize a significant NP decay observed at higher fluence levels; similar fluences are used for soft tissue and cell culture ablations.^16–18^ The stability of the NPs was monitored in the range of 0.2–1.3 J/cm^2^. Signal transients for individual fluences are shown in Figure S4 of the Supporting Information. Only rare decay of NPs during ablation was observed at a fluence of up to ∼1 J/cm^2^; fewer than ∼5% of the detected NPs decay with the exact value, depending on the fluence. When fluences higher than 1 J/cm^2^ were used, most of the NPs decayed. NP decay was observed as an increased signal baseline around individual NP peaks. While transient signal analysis is not the most precise method for assessing NP decay, compared, e.g., to direct sizing of individual NPs and their fragments, it is sufficient for our method as it focuses on excluding false positive counts caused by large NP fragments. Importantly, minor degradation, such as the formation of fragments below the detection limit or partial surface evaporation, does not interfere with NP counting and therefore does not affect the calculated TE. To achieve complete agarose layer ablation using this low fluence, the scan speed was slowed down, resulting in considerable overlap of individual laser pulses and repeated irradiation of each position on the agarose layer. The spot size for 213-nm ablation was set to 100 μm to maintain acceptable analysis times (∼10 min). The TE was determined similarly to the 2940-nm system, with results shown in Table 1. Calculated RSDs for the 213-nm system (4.1%) were greater than the Poisson noise (1.3%).

The NP signal histograms for UCM and both laser ablation systems are shown in Figure S5. The distributions of detected NPs for both the 2940- and 213-nm systems are very narrow, with a maximum of around 220 counts.

#### Assessment of the Remaining Material

We also repeated ablations in the same area to assess the remaining material on the glass using both 2940- and 213-nm LA systems. In the second 2940-nm LA run, less than 1% of NPs initially detected by the first ablation were present. In the case of 213-nm LA, around 6% of NPs were detected during the reablation. Despite visually clean glass after the first 213-nm LA, an increase in reablation yield indicates the presence of a significant amount of unablated material due to mechanical limitations of the *XY* stage in the ablation systems (∼2.5 μm) and redeposited material. Thus, to calculate TE at low 213-nm laser fluence, it is advisible to repeat ablation. The reablation histogram mode (see Figure S5D), which is 208 counts, remains virtually unchanged, compared to the histogram mode from the first ablation (see Figure S5B). This suggests that the NPs were not significantly altered or damaged during either ablation process (both the ablation and reablation within 20 min). The TE calculated from NP counts involving reablation is shown in Table 1.

### Grid Area Approach

Although the method with an unbounded agarose layer area gives practical results, their uncertainties are limited by Poisson statistics and uneven NP distribution in agarose. To avoid the uncertainty due to selecting a random area on the agarose layer, we propose to count NPs on the same, predefined square area by UCM and LA-SP-ICP-MS. Due to the nondestructive nature of UCM, LA-SP-ICP-MS can subsequently examine the same area of an undamaged sample. This approach also allows for a direct comparison of NP distribution within the agarose layer through imaging.

#### 2940-nm LA-ICP-MSI

For imaging, a square in the grid-modified agarose layers was ablated by the IR system under conditions adjusted for imaging; the time between two consecutive laser pulses was set to ∼100 ms, above the ablation cell washout time of ∼75 ms,^19^ to minimize the overflow of NPs into the following pixels. For 213-nm ablation, conditions remained the same as in the case of the random area approach.

Overlaying images with different lateral resolutions, shifts, and rotations with no distinctive features is not straightforward and was accomplished using a special algorithm, as detailed in Figure S6 of the Supporting Information. UCM and 2940-nm LA-SP-ICP-MS maps of a selected square area on the agarose layer are shown in Figure 1. While at the macroscopic level, the images show fine alignment, careful examination reveals an imperfect overlay of UCM-localized NPs with the ICP-MS map. This misalignment can be attributed to NP spillover into adjacent pixels, mostly due to mechanical limitations of the *XY* stage discussed previously. Another possible contribution may be the accumulation of material from the previous line on the edge of the next line; this is also seen on the square edges in Figure 1A. Nevertheless, the spillover of NPs into surrounding pixels does not affect the total number of detected NPs, only their spatial distribution; therefore, the agarose layers can still be used for accurate TE determination. The average detected NP counts for IR-LA and the calculated TE are in Table 1, NP counts from individual experiments can be found in the Supporting Information. By ablating a defined area with a precisely known number of NPs, we eliminated the effect of inhomogeneous NP dispersion in the gel and thus reduced the RSD’s of the method by 3% or 5% for 2940- or 213-nm laser ablation, respectively. The TE value was in excellent agreement with that determined by the random area approach.

**Figure 1:**
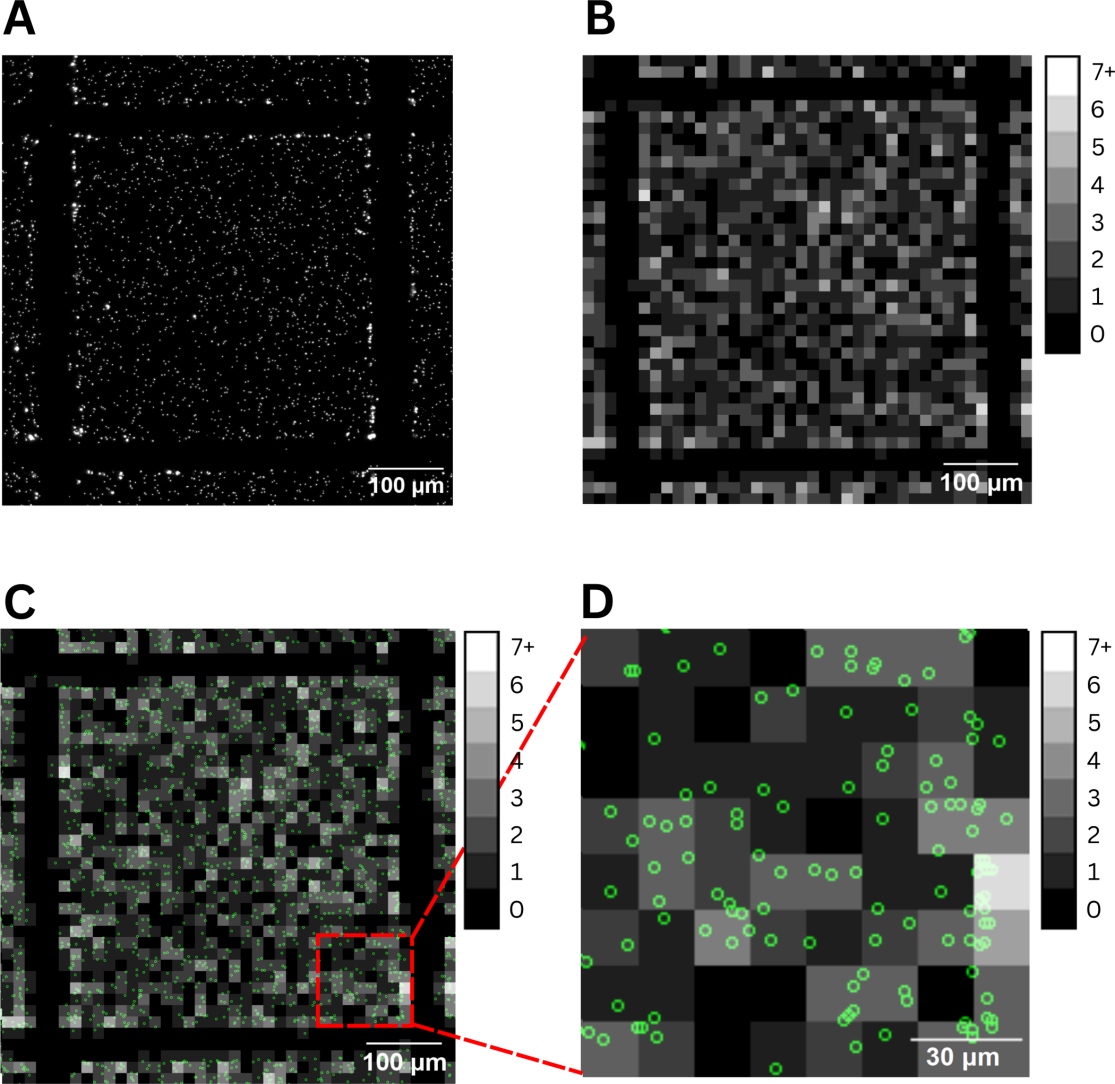
2940-nm LA-SP-ICP-MS and UCM image overlay. (A) UCM image, with bright spots indicating individual NP signals. (B) Digital map of NP distribution measured by 2940-nm LA-SP-ICP-MSI, displayed in grayscale. The shades of gray represent the precise number of NPs in the agarose layer, with a pixel size of 15 μm × 15 μm. (C) Overlay of images A and B, where NPs localized by UCM are highlighted as green circles. (D) A zoomed-in section of panel (C), providing a detailed look at the overlay.

#### 213-nm LA-ICP-MS

No image was generated by the 213-nm system due to the long washout time associated with the commercial ablation cell. The ablation parameters were consistent with those used in the random area approach. Table 1 provides the average NP counts for 213-nm LA from the first ablation and reablation and the corresponding calculated TE or without reablations. Exact NP counts can be found in the Supporting Information.

The calculated TEs for the 213-nm system were slightly lower, but within the experimental error, for the grid approach, compared to the random area approach. This deviation aligns with the observation of higher residual material on the glass substrate after ablation using the grid approach, as evidenced by repeated ablation experiments. The reablation yield for the grid approach was 11%, compared to 5% for the random square approach. Based on these findings; to compare the random square approach and the grid approach for the 213-nm laser, one should consider the yields of ablated NPs and reablations. The overall ablation yields are identical for both approaches (85%). These results emphasize the importance of measuring TE on a daily basis under specific experimental and system conditions to ensure accurate interpretations.

#### Determination of the Remaining Material

In addition to the reablations, the amount of material remaining on the glass after ablation can be estimated by performing repeated UCM of the same site and comparing the number of NPs before and after ablation. This process was also used to determine the effectiveness of reablations. Detailed image comparison is available in Figure S7 of the Supporting Information.

For the IR system, only units of NPs were detected by UCM after ablation over the entire ablated area. After 213-nm ablation, the number of remaining NPs on the glass was significantly higher compared to the IR system. This is a direct consequence of the narrow fluence window for the release of intact NPs in the 213-nm system. The number of remaining NPs detected on the glass after the first ablation was up to 15% of the original number detected by UCM in agreement with ICP-MS counts from the first ablation and reablation. After reablations, this number decreased to values similar to those for the IR system after the first ablation, and the numbers of remaining NPs were in the order of units over the entire reablated area.

#### 193-nm-LA-ICP-MS

To examine the 193-nm ablation system coupled to the quadrupole ICP-MS, three scan lines covering an area of 13 mm^2^ were performed. As evident from transient signals in Figure S8A in the Supporting Information, the ablation process leads to an elevated baseline of the transient signal, particularly around clusters of NP peaks. This points to partial or complete evaporation of NPs during the ablation process. In an additional experiment, we also coupled the 193-nm ablation system with an SP-ICP-TOFMS and investigated areas of 0.5 mm × 0.5 mm, using both random square and grid approaches. Despite reduced sensitivity of TOF mass analyzer relative to the quadrupole analyzer, the capability to detect more than one isotope per NP and analyze NP composition can be seen as a critical advantage in future applications. Not only does it enable a nontarget analysis in uncharacterized samples but it also further promotes rapid multielement imaging and, therefore, multiplexed mapping. This could, for example, be advantageous in digital mass spectrometry as well as mass cytometry imaging, where NPs are considered as antibody labels. The reduced sensitivity for single isotopes in SP-ICP-TOFMS may pose a challenge because small particles transported into the ICP may be below the critical limit and be missed. The false negative detection rate (beta error) however would be size-dependent and be more expressed for small particles. Therefore, in the case of small NPs, TEs could be underestimated significantly. However, for sizes used in the presented experiments, TEs were comparable to quadrupole-based instruments and the beta error was not increased significantly. Signal transient similar to that observed with the quadrupole ICP-MS and corresponding histogram confirm significant NP disintegration as well (see Figures S8B and S8C in the Supporting Information). Furthermore, due to the higher inherent noise and elevated detection threshold of the TOF-MS, smaller NP peaks might not be counted. The estimated TE of the 193-nm laser ablation systems was significantly lower (∼43% for the quadrupole MS and ∼42% for the TOFMS), compared to the 213-nm (85%) and 2940-nm (95%) laser ablation systems. This difference underscores the necessary caution required when applying this approach for the determination of TE using laser ablation at shorter wavelengths. Absorption of common materials at 193-nm is generally higher than at 213-nm,^26^ resulting in harsher ablation conditions that result in NP loss. It is evident that all NPs desorbed by the 193-nm laser cannot be counted, and the calculated TE is subject to negative systematic error.

## Conclusion

This study presents a direct method for determining TE in LA-SP-ICP-MS. The TE is calculated from the numbers of NPs released from agarose layers, which were previously characterized by UCM. The agarose layers with a specific number of NPs can be prepared, characterized, and archived for future use as a storage-stable standard. This study provides valuable insights into the laser ablation of Y-based upconversion NPs, highlighting wavelength-specific influence on their integrity and transport. The observed differences in NP integrity and transport reflect the behavior of these particular NPs in the employed laser ablation systems and may not directly apply to other NP types and sizes.

With the 2940-nm laser, the method was robust and reliable, achieving near-quantitative NP desorption. Yet, reablation is advisible to confirm the proper adjustment of the laser ablation system. The TE for this wavelength was primarily determined by the design of the ablation cell and the efficiency of aerosol transport. The 213-nm laser, while effective, required careful optimization of fluence to mitigate incomplete desorption and possible NP redeposition, which introduced variability in TE measurements. In contrast, the 193-nm laser showed significant limitations due to NP disintegration, elevated baseline, and noise, leading to a reduced TE. A possible remedy to NP disintegration could be using a poly­(methyl methacrylate) or poly­(vinylpyrrolidone) matrix for NP immobilization,^22^ probably due to stronger absorption at 193-nm. We also tested the application of a TOF instrument, which potentially could miss small particles due to its reduced duty cycle. However, for NP sizes used here, we could demonstrate comparable TEs. This might be interesting for future studies as LA-SP-ICP-TOFMS would allow significantly faster mapping and a multiplex analysis, which are critical factors for many biochemical and/or medical applications (e.g., imaging mass cytometry).

This work emphasizes tailoring TE assessment methods to the unique characteristics of each LA system. By addressing challenges such as particle loss and signal variability, this method lays foundations for improved NP characterization and LA imaging across diverse applications. Future research should focus on optimization of this approach for systems operating at shorter wavelengths, which are the most used in the ICP-MS applications nowadays. Additionally, strategies to minimize particle loss and improve signal processing will be crucial for optimizing TE and broadening the applicability of this method.

## Supplementary Material



## Data Availability

The data underlying this study are available in the data repository at https://data.narodni-repozitar.cz/general/datasets/hcr8t-k8t55 (DOI: 10.48700/datst.hcr8t-k8t55).

## Notes

Detailed nanoparticle synthesis procedures, gel preparation methods, and silica shell coating; experimental setup for laser ablation and ICP-MS systems; full UCM imaging methodology; data evaluation algorithms and settings for both quadrupole and TOF ICP-MS; signal transients and histograms at varying laser fluences; image alignment algorithm for UCM and LA-ICP-MS; UCM verification of residual material after ablation; and additional results from the 193-nm LA-SP-ICP-MS system (PDF)
