# Supplementary material for: A Method for Determination of Transport Efficiency in Laser Ablation Inductively Coupled Plasma Mass Spectrometry for Tissue Analysis
Source: Anal Chem. 2025 Jun 15;97(25):12940–6. doi: 10.1021/acs.analchem.5c01306 (PMC12224154; doi:10.1021/acs.analchem.5c01306)
Supplement: Supplementary file 1 [file ac5c01306_si_001.pdf]

Supporting Information

A method for determination of transport efficiency in laser ablation inductively coupled plasma mass spectrometry for tissue analysis

Jaromír Stráník[1], Vilém Svojanovský[1], Julie Weisová[2], Kateřina Uhrová[2], David Clases[3], Antonín Hlaváček[2*], and Jan Preisler[1*]

[1]Department of Chemistry, Faculty of Science, Masaryk University, 625 00 Brno, Czech Republic

[2] Institute of Analytical Chemistry of the Czech Academy of Sciences, 602 00 Brno, Czech Republic

[3] NanoMicroLab, University of Graz, 8010 Graz, Austria

Corresponding authors:

Jan Preisler

Department of Chemistry, Faculty of Science, Masaryk University

625 00 Brno, Czech Republic

ORCID: 0000-0002-9819-1284

email: preisler@chemi.muni.cz

Antonín Hlaváček

Institute of Analytical Chemistry of the Czech Academy of Sciences

602 00 Brno, Czech Republic

ORCID: 0000-0003-3358-3858

email: hlavacek@iach.cz

Table of Contents

Nanoparticle synthesis S-3
DLS characterization of UCNPs S-9
Gel layer preparation S-7
Upconversion microscopy S-8
ICP-MS S-10
Nanoparticle disintegration as a function of laser fluence S-12
Algorithm for alignment of ICP-MS and optical digital maps S-15
Determination of remaining material after ablation and re-ablation S-16
Results of 193-nm LA-SP-ICP-MS S-17
References S-19

Nanoparticle synthesis

Chemicals

Yttrium(III) oxide (99.99% trace metals basis), ytterbium(III) oxide (99.99% trace metals basis), thulium(III) oxide (99.99% trace metals basis), yttrium(III) chloride hexahydrate (99.99% trace metals basis), ytterbium(III) chloride hexahydrate (99.99% trace metals basis), thulium(III) chloride hexahydrate (99.99% trace metals basis), sodium oleate (≥82% fatty acids, powder), ammonium fluoride (ACS reagent,>98%), 1-octadecene (technical grade, 90%), polyoxyethylene (5) nonylphenyl ether (igepal CO-520), tetraethyl orthosilicate (TEOS, ≥99.0%), oleic acid (technical grade, 90%), Na_2CO_3 (ACS reagent, ≥99.7%), were from Sigma/Merck. Methanol (>99.8%), cyclohexane (>99.5 %), propan-2-ol (p.a.), ammonia solution (25%, p.a.), acetone (p.a.), and N,N-dimethylformamide (DMF, >99.5%) were from Penta. Trifluoroacetic acid (≥99.9 %) was from Roth. Carboxyethylsilanetriol (CEST) sodium salt (25%) in water was from abcr.

Rational design of lanthanide-doped photon-upconversion nanoparticles

The composition and architecture of lanthanide-doped upconversion nanoparticles (NPs) were designed to achieve high photon-upconversion (UC) efficiency, high yttrium content detected by mass spectrometry), and good reproducibility of synthesis. However, optimizing the NP formulation is inherently challenging, as UC efficiency is governed not only by the dopant ions but also by their concentration, spatial distribution within the NP (i.e., core-only versus core-shell configurations), host composition, and the excitation intensity. Among known systems, two formulations exhibit particularly high UC performance: $NaYF_4$ nanocrystals doped with ~20 mol% Yb^{3+} and either ~2 mol% Er^{3+} or ~2 mol% Tm^{3+}. For Er^{3+}-doped NPs, it is common practice to add an undoped $NaYF_4$ shell around the Er^{3+} -doped core to suppress surface-related quenching and enhance the luminescence. A drawback of core-shell architectures is the significant increase in overall particle size. For instance, a quantum yield (QY) of 9% was reported for 45-nm Er^{3+}-doped NPs with a thick $NaYF_4$ shell (core-to-shell volume ratio of 1:7) under 40 W cm² excitation.[1] However, only ~12.5% of the NP volume was luminescent (the core itself). In contrast, core-only Tm^{3+}-doped NPs with a diameter of ~30-nm achieved a QY of 8.4% under 140 W cm² excitation, demonstrating that high UC efficiency is attainable without the need for a passivating shell.[2] Furthermore, Tm^{3+}- and Er^{3+}-based NPs exhibit distinct emission profiles. Er^{3+}-doped NPs typically emit at both ~550-nm and ~650-nm, resulting in reduced signal when a band-pass filter is applied to minimize chromatic aberration of the microscope. In contrast, Tm^{3+}-doped NPs predominantly emit around ~800-nm (under the excitation conditions used for microscopy imaging in this study), enabling more efficient signal detection when applying a band-pass filter. In summary, Tm^{3+}-doped core-only NPs were selected as the optimal nanomaterial for investigating transport efficiencies because of their high upconversion efficiency, high yttrium content, and simple core-only architecture, resulting in good synthesis reproducibility.

Preparation of trifluoroacetates for growth of seed nanoparticles

Under reflux, Y_2O_3 (1806.5 mg, 8.0 mmol), Yb_2O_3 (709.3 mg, 1.8 mmol), and Tm_2O_3 (77.17 mg, 0.2 mmol) were dissolved in trifluoroacetic acid (10 mL) and water (10 mL) in a 250 mL three-

necked flask. When dissolved, Na_2CO_3 (1059.88 mg, 10 mmol) was added, releasing bubbles of CO_2 and dissolving rapidly to a clear solution. After removing the condenser, excessive trifluoroacetic acid and water were evaporated by heating at 110 °C in a fume hood. The resulting white powder of trifluoroacetate was dissolved in oleic acid (22.5 mL, 20.1 g) and 1-octadecene (22.5 mL, 17.8 g). Then, sodium oleate (917.89 mg, 3.25 mmol) was added. This solution was diluted with 20 mL of methanol. The methanol, together with oxygen and water, was removed by heating at 110 °C for 20 minutes under an inert N_2 atmosphere. The resulting precursor solution was enclosed in the flask by silicon septa and kept under an inert atmosphere. To decrease the viscosity, the precursor solution was kept at an elevated temperature (~50 °C), which facilitated its injection into a hot reaction mixture. The concentration of $Re(CF_3COO)_3$ in the precursor solution was 0.17 mmol mL^{-1} (Re for Y, Yb, and Tm in molar percentages of 80%, 18%, and 2%, respectively).

Synthesis of seed nanoparticles

$YCl_3 \times 6\ H_2O$ (1213.4 mg, 4 mmol), $YbCl_3 \times 6\ H_2O$ (348.74 mg, 0.9 mmol) and $TmCl_3 \times 6\ H_2O$ (38.34 mg, 0.1 mmol) were dissolved in methanol (30 mL) and added into a 250 mL three-neck round-bottom flask containing oleic acid (27.5 mL) and 1-octadecene (85 mL). The solution was heated to 170 °C under an N_2 atmosphere for a time long enough to remove all volatile liquids and then cooled to 50 °C. Then, the protective atmosphere was disconnected, and the solution of NH_4F (740.8 mg, 20 mmol) and NaOH (500.0 mg, 12.5 mmol) in methanol (40 mL) was added to the intensively stirred solution. The N_2 atmosphere was reconnected, and the solution was stirred for 60 min. The temperature was carefully increased to 150 °C, avoiding extensive boiling to ensure the evaporation of methanol. After that, the solution was rapidly heated at the rate of ~10 °C per minute. At 290 °C, the heating was carefully regulated to 300 °C within one or two minutes. The flask was kept under N_2 flow at 300 °C for 90 min. Finally, the flask was cooled to room temperature.

Growth of seed nanoparticles

The reaction mixture of volume 22.5 mL containing the seed NPs was transferred into a 100 mL three-necked round bottom flask with 15 mL of methanol. The NPs were grown by gradually adding the precursor solution to the solution of seed NPs. The mixture was heated at 150 °C for ~30 min under the nitrogen atmosphere to remove oxygen and water. Then, the temperature was rapidly increased to 300 °C. Keeping this temperature, a syringe with a 120-mm long needle was used to inject the precursor solution ten times (respective volumes: 2.7, 3.2, 3.7, 4.4, 5.0, 5.7, and 6.8 mL), each with a delay of 10 min. After the last addition, the temperature was kept at 300 °C for 10 min, eventually preparing NPs of the desired size (reaction mixture volume ~54 mL). The resulting NPs were precipitated by adding propan-2-ol (190 mL) and collected by centrifugation (1,000g, 10 min). The pellet was washed with 30 mL of methanol, and the NPs were shortly sonicated and centrifuged (1,000g, 30 s). The pellet was extracted three times with 18 mL of cyclohexane. Each extracted aliquot was centrifuged (50g, 20 min) to separate coarse grains from the dispersion. Finally, all aliquots were combined, and the final product was stored at laboratory temperature.

Estimating the mass concentration of nanoparticle dispersion

For estimating NP mass concentrations, the volume of 250 µL of oleic acid-capped NPs dispersed in cyclohexane was filled into a glass vial. The vial was placed firstly on a heater to evaporate cyclohexane and secondly for 90 min into a furnace at 550 °C. The mass of NPs was then used to calculate the mass concentration.

Synthesis of the carboxylated silica shell

NPs (30 mg) were coated with a carboxylated silica shell in a microemulsion. NPs were diluted in cyclohexane (to the final volume of 15.3 mL) with Igepal CO-520 (900 mg) and tetraethyl orthosilicate (51 µL) and stirred with high intensity for 10 min. The microemulsion was created after adding an aqueous ammonium hydroxide (12% w/v, 113 µL). The resulting mixture was slowly stirred for 18 hours. Another volume of tetraethyl orthosilicate (13 µL) was added, and the microemulsion was slowly stirred for 4 hours. Carboxyethylsilanetriol sodium salt (25% w/v in water, 26 µL) was added, and the cloudy emulsion was sonicated for 15 min and further stirred for 60 min. Carboxylated NPs were extracted by adding DMF and washed three times with acetone (4.5 mL) and three times with water (4.5 mL). Carboxylated NPs were finally dispersed in 3 mL of water and stored at 4°C.

DLS characterization of UCNPs

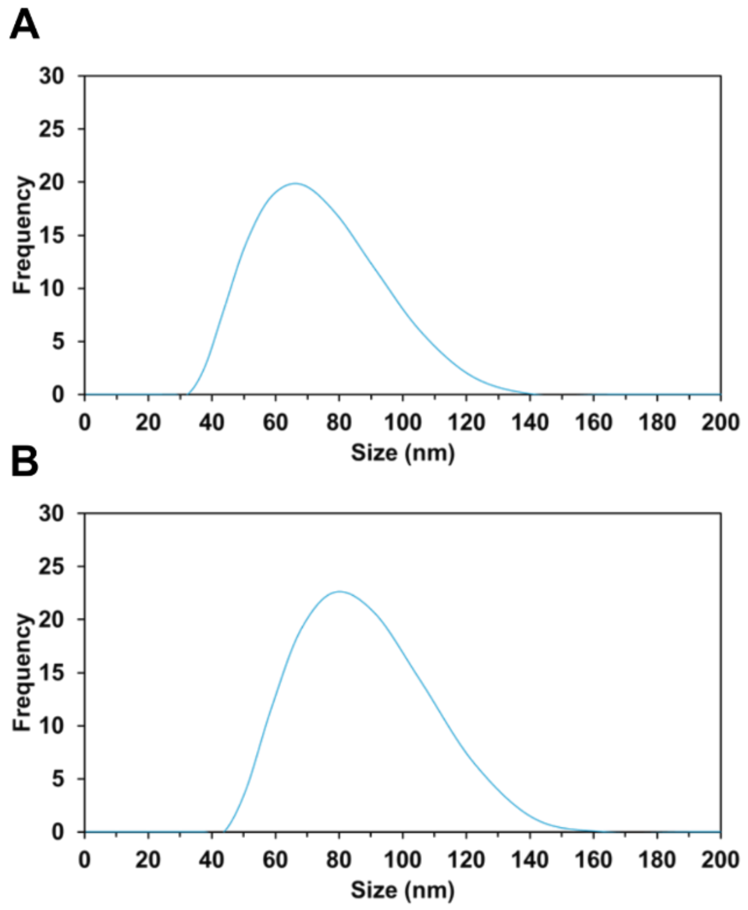

Figure S1. Size distribution of UCNPs without (A) or with (B) silica shell measured by DLS. The particle size without silica shell (A) was 69 ± 19 nm and with silica shell (B) 83 ± 20 nm.

Gel layer preparation

Agarose was selected as the immobilization matrix because it provides an optically clear and hydrophilic environment suitable for UCM imaging. Moreover, agarose-based gels are commonly used as matrix-matched calibration standards in laser ablation ICP-MS for tissue analysis due to their structural similarity to biological samples and ability to form homogeneous, reproducible layers upon drying. To strengthen the point that the prepared agarose gels are useable as a standard for TE determination of soft tissues, we also performed a simple verification experiment, in which 200 nL of the same UCNP suspension was applied to an agarose gel and simultaneously to a cryosection of mouse brain tissue using a syringe. Then, the droplets were ablated by the 2940-nm laser system, and the number of detected particles was compared. On average, 4400 ± 300 (average ± SD, n=3) were detected from agarose and 4100 ± 700 (average ± SD, n=3) from tissue. The averages were not statistically significantly different at the 0.05 significance level according to Lord's test. Histogram modes were 140 ± 5 (average ± SD, n=3) for agarose and 129 ± 6 for tissue; the experiments were carried out on different days.

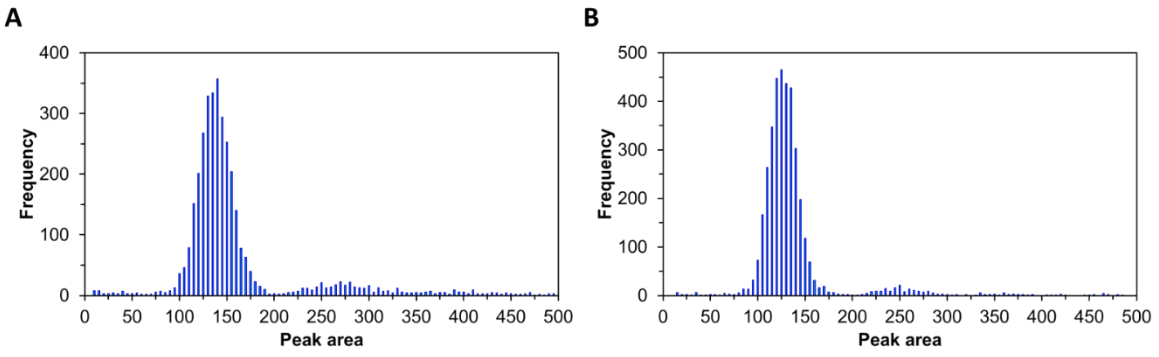

Figure S2. 2940-nm LA-SP-ICP-MS histograms from (A) agarose gel with a histogram mode of 130 counts and (B) brain tissue with a histogram mode of 125 counts.

The immobilization of NPs into the agarose submicron layer

The dispersion of NPs was diluted with a dispersion of melted agarose in water kept at 60 °C 1.25% w/v (NEEO ultra-quality agarose, Carl Roth, Germany). The resulting dispersion was cast as a 38 ± 1.2 µm layer between two glass slides separated with plastic tape. After 15 min in the refrigerator (4 °C), the cover glass was removed, and the agarose gel dried rapidly (anisotropic collapse), forming a homogeneous submicron layer with NPs as reported previously.[1,2] The glass slide with the agarose layer was enclosed in a Petri dish to protect it from dust and stored at laboratory temperature.

Agarose layer modification

To ensure consistent targeting of the same area with both UCM and LA-ICP-MS, square regions measuring approximately 0.5 mm × 0.5 mm were created by ablating grid using an optical parametric oscillator (OPO) Opolette 2940 with emission wavelength of 2940-nm (Opotek, Carlsbad, CA), with a fluence of 42 J/cm^2 and spot size of 45 µm, leading to an equally broad gap between adjacent squares. During the ablation, the agarose layer was rinsed with a

continuous stream of air to prevent the material ablated by the laser from settling around the laser-created traces. The laser ablation system employed for the agarose layer modification will be described later.

Upconversion microscopy

A laboratory-built epiphoton-upconversion microscope was used, see Figure S2. A 10 W fiber-coupled laser diode with an emission wavelength of 976-nm (Roithner Lasertechnik, Wien, Austria) was used for NPs excitation. A lens projected the laser beam from the laser diode onto the customized rotating diffuser. The image of the laser spot on the diffuser was projected by the additional lens into the back focal plane of the microscope objective - Köhler illumination. Before entering the microscope dichroic mirror with a short-pass 900-nm (Thorlabs, Newton, NJ), the laser beam was passed through a long-pass optical filter with a cut-off wavelength of 925-nm (Thorlabs, Newton, NJ). The microscope camera was protected from excitation wavelengths by an 875-nm short-pass filter (Edmund Optics, Barrington, NJ). An 800 ± 25 nm band-pass filter (Edmund Optics, Barrington, NJ) was used for selecting only the near-infrared emission of NPs. An air immersion microscope objective CFI BE2 Plan with 20× magnification and a numerical aperture of 0.40 (Nikon, Tokyo, Japan) was used for NP imaging. The laser power illuminating the sample was 2.4 W, measured by a microscope slide thermal sensor (Thorlabs, Newton, NJ) placed in front of the microscope objective. The micrographs were recorded by EM-CCD camera iXon Life 888 (Andor Technology - Oxford Instruments, Belfast, United Kingdom). The camera was carefully calibrated to compensate for laser illumination irregularities, variations of the sensitivity between different pixels, and the background.

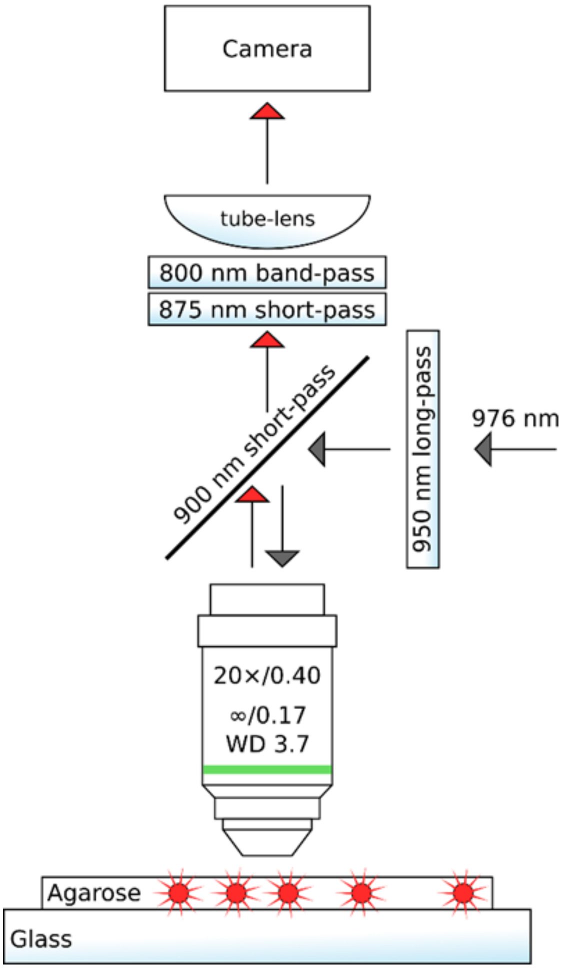

Figure S3. Optical setting. The excitation laser (grey arrows) goes through a long-pass filter to remove shorter wavelengths from a laser beam. A short-pass dichroic mirror reflects the laser beam through a microscope objective onto the NPs (red stars), immobilized in the anisotropically collapsed agarose gel on a glass substrate. The microscope objective collects the emission from NPs (red arrows). The short-pass filter protects the camera from excitation wavelengths, and the band-pass filter passes only the near-infrared emission of NPs. A tube lens projects the emission into the camera.

The UCM data evaluation

FIJI-ImageJ was used for manual micrograph processing. For automatic localization and counting of NP spots in micrographs, a convolutional network with a U-net architecture was utilized as reported previously[3-5] (implemented in the Python version 3.10 programming language using the Keras version 2.15 deep learning interface). After localization, the emission intensities of NP spots were measured by integrating the signal of each spot in the circular area with a diameter of 6 pixels (3.9 µm in the sample plane). The intensity was not measured for spots separated by less than 10 pixels (6.5 µm) from each other to avoid the cross-talk of the emission between close spots. The background signal was measured by integrating the signal in the randomly positioned circles with a diameter of 6 pixels (3.9 µm) and at least 15 pixels (9.8 µm) from the closest spot. Upon subtracting the background, the histogram of spot intensities was constructed.

ICP-MS

An ICP quadrupole mass spectrometer Agilent 7900 (Agilent Technologies, Santa Clara, CA) was combined with 2940, 213, and 193-nm laser ablation systems. The 193-nm laser ablation system was also coupled to the Vitesse ICP-TOF mass spectrometer (Nu Instruments, UK).

The 2940-nm laser ablation system was linked to the quadrupole ICP mass spectrometer through a 1.34-mm inner diameter stainless steel capillary transport tube (Swagelok, Solon, OH) via a low-volume laser ablation adapter from Glass Expansion (Port Melbourne, Australia) with a make-up gas Ar flow rate of 0.4 L/min to introduce the dry aerosol into the ICP. A quartz plasma torch with a 2.5-mm injector width (AHF analysentechnik AG, Tübingen, Germany) was employed to sustain a total gas flow rate of 2.0 L/min. The ICP-MS was operated in a time-resolved analysis mode with the shortest dwell time possible, 100 µs, to minimize errors from partial NP event integration and coincidental events. The isotope of choice detected within the NPs was ^{89}Y, as it had the highest abundance. We used yttrium standard solution (Analytika, Prague, Czech Republic) diluted to 1 µg/L to adjust the position of the plasma torch and voltage of the ion optics and maximize the detector's response at the mass-to-charge ratio (*m/z)* 89.

For the 213-nm LA, the same MS setup was used as in the 2940-nm LA-ICP-MS, with a minor change to tubing and Ar flow rate. The connection between the ablation cell and the plasma torch was facilitated by a 2.5-mm inner diameter polyurethane tube (Parker Legris, Cleveland, OH), and the 1.1 L/min Ar make-up gas was introduced via a quick-connect.

For the 193-nm LA, the quadrupole system was connected to the laser using a Fluorinated Ethylene Propylene (FEP) tube with an inner diameter of 2 mm. Gas flow rates in the ablation cell were configured to match those used for the 213-nm laser ablation system. The quadrupole mass spectrometer settings were identical to those used for the 213-nm measurements. In the case of LA-ICP-TOFMS, the laser was coupled to the mass spectrometer via an aerosol rapid introduction system (ARIS) for aerosol transport. Parameters such as Ar flow, torch position, and TOF parameters were optimized daily to obtain the best sensitivity by ablating the NIST 612 "Trace Elements in Glass" reference material to maximize the response for ^{115}In, ^{238}U, and ^{89}Y. Final measurements were conducted over a reduced mass range of 39–89 amu with a 76.89 µs dwell time.

ICP-MS data evaluation

Data from the LA-SP-ICP quadrupole MS underwent processing using a dedicated program "Analyzer" developed within the LabVIEW 2017 environment. The analysis of data recorded via LA-SP-ICP-TOFMS required the application of compound Poisson statistics, and all data treatment, processing, and visualization was done in the open-source SP analysis platform "SPCal" (version 1.2.10) developed by Lockwood et al.[6,7] using an alpha value of 10^{-6}.

The primary functionalities of the software "Analyzer" include rendering intensity maps, NP count maps, signal intensity profiles for selected lines or pixels, and histograms for analyzing peak count and peak area. The input data comprises a series of files, each representing a time record of ion signal intensities at *m*/*z*=89 with a 100 µs integration time from a single image line. Initial data filtration was based on a specified threshold and minimal peak width. Once

all peaks were identified, their position on the time axis, amplitude, and area were determined. Peaks exceeding the threshold for a period longer than the pre-set maximum peak width were segmented into multiple parts, treating each segment independently as an individual peak. In such cases, NP counts could be distributed between adjacent pixels.

A histogram of peak areas was constructed and fitted with a log-normal distribution. The mode, representing the most probable peak area, was determined. The NP count in each peak was determined from the ratio of the peak area to the mode. Subsequently, the peak detection parameters for 69-nm NPs were set as follows: threshold of 3 counts was selected above the baseline noise, minimal peak width: 2 pts, maximal peak width: 6 pts, so all the signal events between 200 – 600 µs were chosen as a signal of an NP. To exclude small NPs and NP aggregates, only NPs in peaks presumably containing 1 to 3 NPs (i.e., with the ratio of the peak area to the histogram mode between 0.5 and 3.5) were counted.

Nanoparticle disintegration as a function of laser fluence

The signal transient of NPs desorbed using the 2940-nm laser ablation system at a fluence of 42 J/cm² in Figure S3A reveals well-defined peaks corresponding to intact NPs with a minimal baseline, indicating negligible vaporization or fragmentation. A detailed view of the baseline in Figure S3B confirms the virtual absence of noise, suggesting that most NPs remain intact during ablation at this laser fluence.

To test the NP disintegration in the 213-nm laser ablation system, we ablated the gels at a laser fluence of 0.2, 0.6, and 1.3 J/cm². At low laser fluence (0.2 J/cm²), the signal transient (Figure S3C) displays individual, well-defined peaks corresponding to intact NPs. A detailed view of the baseline (Figure S3D) highlights minimal noise between NP peaks, indicating a limited extent of NP vaporization or fragmentation. The character of noise proves that the Au atoms released from NPs barely exceed the digitizer noise.

At moderate laser fluence (0.6 J/cm²), the signal transient (Figure S3E) shows a slight increase in noise around the NP peaks, suggesting the onset of NP fragmentation. The baseline detail (Figure S3F) emphasizes this more clearly. At high laser fluence (1.3 J/cm²), the signal transient (Figure S3G) exhibits an elevated baseline reflecting substantial NP damage. The detailed baseline view (Figure S3H) shows a release of atoms and smaller peaks of secondary particles, which underscores the extensive disruption caused by high laser fluence. This progression emphasizes the need to select a proper laser fluence to balance efficient ablation and preserve NP structural integrity.

Smaller peak intensities for the 2940-nm laser ablation system compared to the 213-nm one can be attributed to different calibrations of the ICP-MS (reduced sensitivity on the day of the 2940-nm measurement). Also, differences in gas flow rates and the setup of the two laser ablation systems contribute to the observed variations in peak intensities.

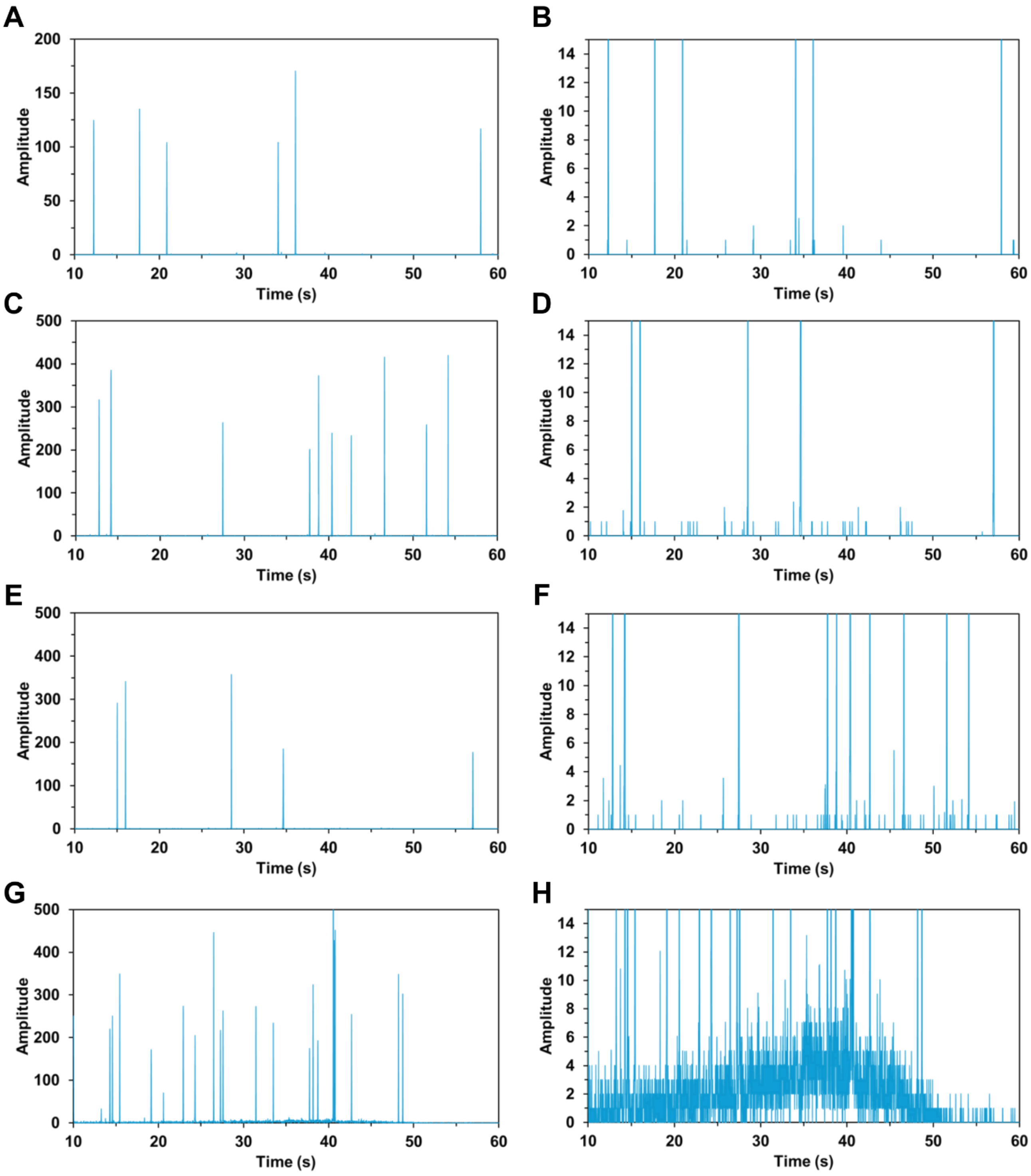

Figure S4. One-minute signal transients (*m/z* 89) recorded during 2940-nm and 213-nm LA-SP-ICP-MS analysis of UCNPs at laser fluence 42.0 J/cm² (A,B) for 2940-nm laser and 0.2 (C,D), 0.6 (E,F), and 1.3 J/cm² (G,H) for 213-nm laser.

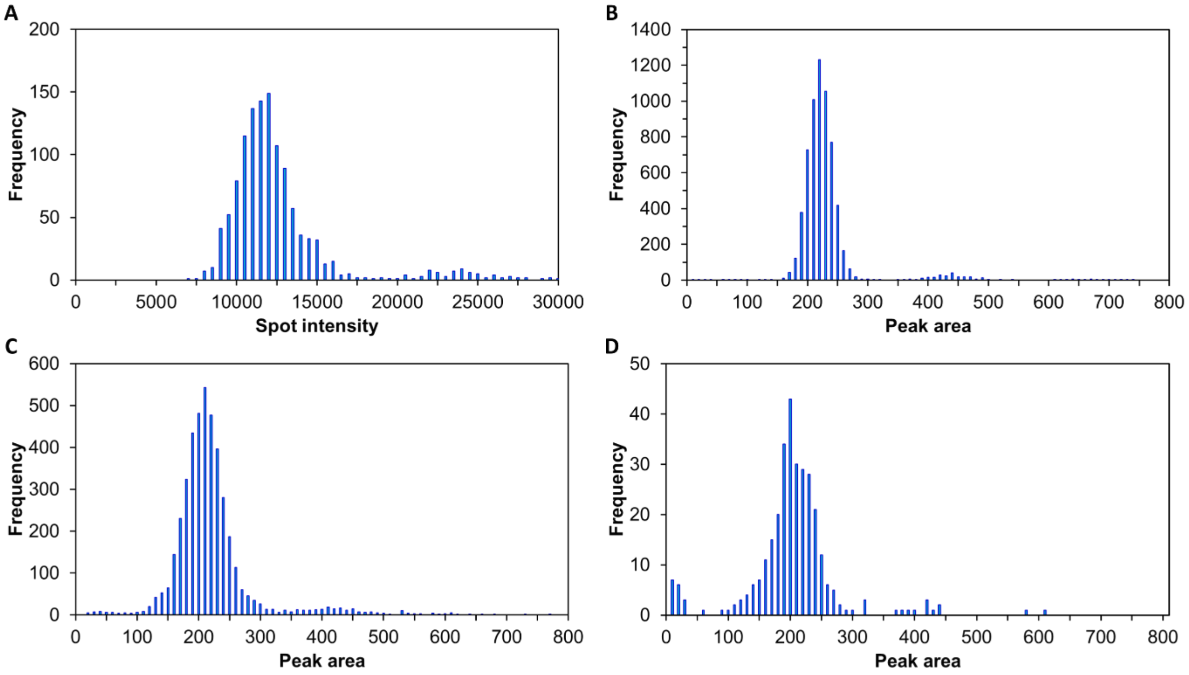

Figure S5. NP histograms. (A) UCM intensity histogram from a random area of 0.667 mm × 0.667 mm on the agarose layer. (B) 2940-nm LA-SP-ICP-MS peak area histogram from a random 1 mm × 1 mm area on the agarose layer, with a mean value of 220 counts. (C) 213-nm LA-SP-ICP-MS peak area histogram from a random area of 1 mm × 1 mm on the agarose layer, with a mean value of 215 counts (D) 213-nm laser re-ablation SP-ICP-MS peak area histogram constructed from 321 remaining NPs. The mean value of 208 counts remains nearly unchanged compared to the histogram from the first laser ablation, suggesting that the NPs were not significantly altered or damaged during both ablation processes. Both ablation and re-ablation were carried out within 20 min.

Table S1 NP counts from first ablation and re-ablation in 2940 and 213-nm laser ablation system using Grid approach

Laser	1st ablation	Re-ablation	UCM
2940-nm	1268	3	1333
	1261	1	1328
	1278	1	1375
213-nm	1085	137	1456
	1056	185	1440
	1085	69	1440

Algorithm for alignment of ICP-MS and optical digital maps

Algorithm overview

To evaluate the accuracy of ICP-MS digital maps, the ICP-MS digital map and the optical micrograph of the same area were recorded. The optical micrograph was then transformed into an optical digital map of the same size as the ICP-MS map. To compensate for the inevitable misalignment of both imaging techniques, the algorithm systematically searched the space of shifts and rotations of the micrograph, finally providing the optical digital map of the highest similarity with the ICP-MS digital map; see Figure S5 for a better understanding. Firstly, a center of coordinates was placed into the center of the optical micrograph (Figure S5A). The positions of all NPs relative to this center were extracted from the image. An empty digital map was aligned with the image center (Figure S5B). The NP positions were shifted and rotated (Figure S5C). After setting new NP positions, the empty digital map was filled with numbers of NPs projecting to its pixels (Figure S5D). A similarity score was calculated for this optical digital map. The similarity scores were calculated for many shifts and rotations, which were systematically generated. The shifts and rotation providing the smallest similarity score were considered optimal (Figure S5 D,E).

Similarity score

The similarity score (*S*) was calculated as a sum of absolute values (*abs*) of differences between the respective pixels of the ICP-MS digital map (ICPMS_px) and the optical digital map (Opt_px); both digital maps contained *N* pixels.

$$S = \sum_{i=0}^{N} abs(ICPMS\_px_i - Opt\_px_i)$$

Searching the space of shifts and rotations

To search the space of shifts and rotations systematically, the score function was calculated for all variations of shifts in the *x*- and *y-axes* and rotations *α*. Symmetrically around the center of coordinates, there were 40 shifts in the *x-axis* and 40 shifts in the *y-axis* with a step size of 2 µm and 160 rotation steps with a step size of 0.05°, resulting in 256000 variations. After finding the shift and rotation with the lowest score, the next search was conducted symmetrically around the best solution from the previous iteration. The numbers of steps in the *x*- and *y-axes* and rotations *α* were kept the same, but the step sizes were reduced by half. Four more iterations were conducted, finally providing the optimized solution.

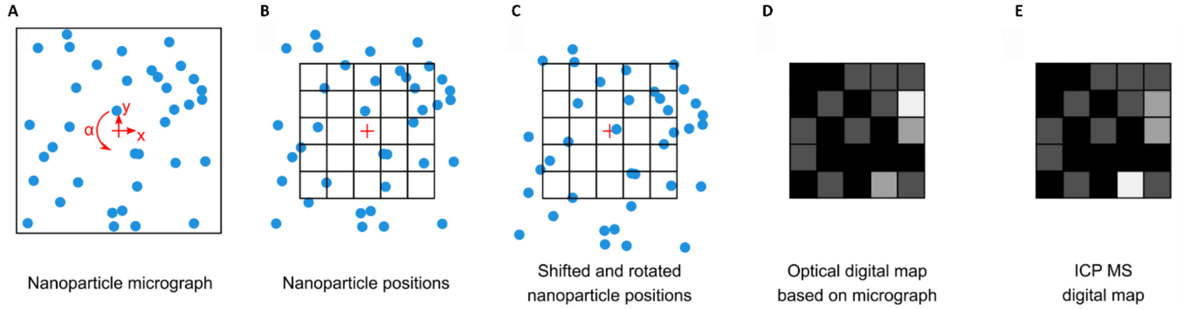

Figure S6. An outline of the optimization algorithm. (A) The center of coordinates and rotation (in red) is placed into the center of the micrograph (gray rectangle), and the positions of NPs (blue dots) relative to the center are extracted. (B) An empty digital map is aligned with the center of coordinates. (C) NP positions are shifted around the *x*- and *y-axes* and rotated around the center. (D) The numbers of NPs are projected to the respective pixels, generating the optical digital map. (E) The digital ICP-MS map can differ slightly from the optical digital map even after optimal alignment.

Determination of remaining material after ablation and re-ablation

UCM was used to monitor NPs remaining after ablation and re-ablation of samples with 213 and 2940-nm lasers. Figure S6A shows an unablated sample with bright spots corresponding to individual NPs. Approximately 15% of the NPs remained after ablation with the 213-nm laser, see Figure S6B. In the case of ablation and re-ablation, fewer than 1% of NPs remained, as seen in Figures S6C,D. The first 2940-nm ablation removed more than 99% of NPs, see Figures S6 E,F.

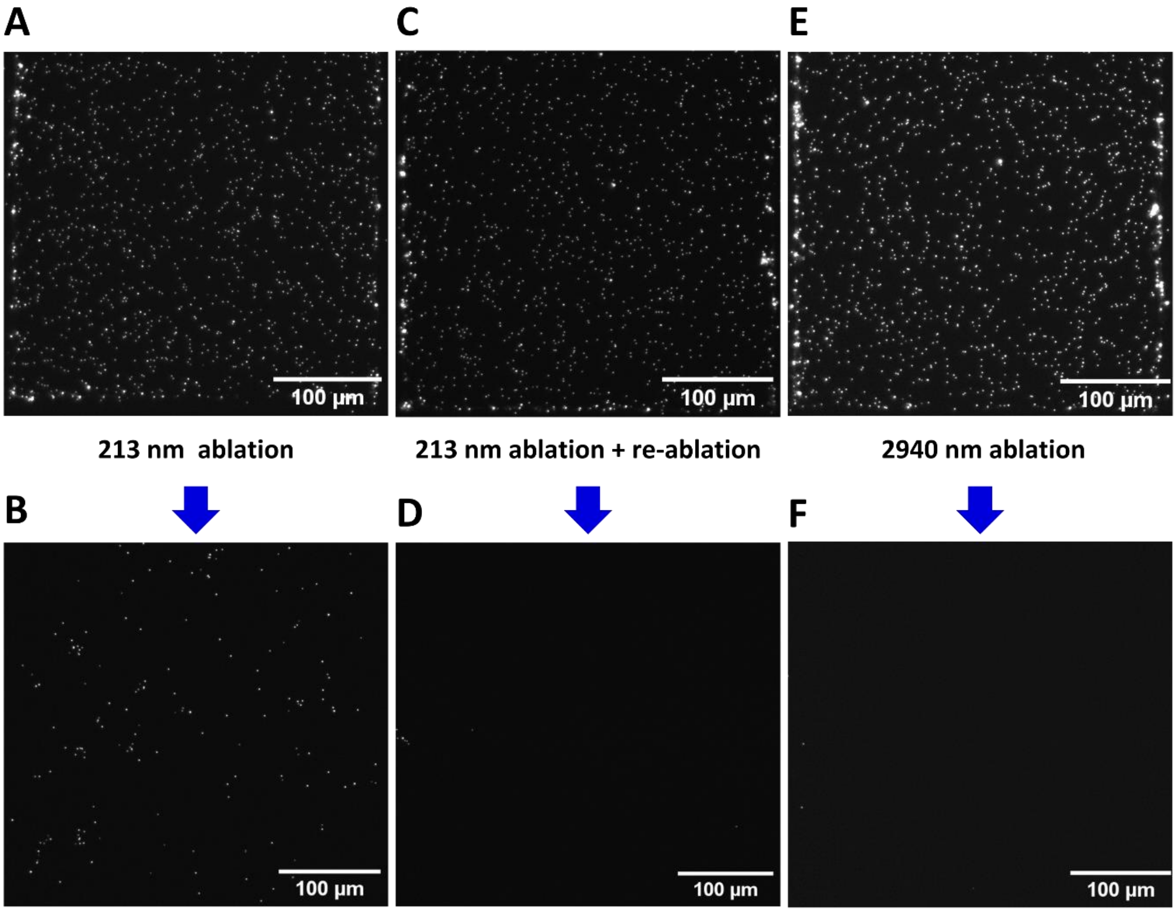

Figure S7. UCM images of a 0.5 mm × 0.5 mm grid area (A) before and (B) after 213-nm ablation. UCM images of a second 0.5 mm × 0.5 mm grid area (C) before 213-nm ablation and (D) after 213-nm laser ablation and re-ablation. UCM images of a third 0.5 mm × 0.5 mm grid area (E) before and (F) after the first 2940-nm laser ablation.

Results of 193-nm LA-SP-ICP-MS

For the testing of a 193-nm laser ablation system coupled to the quadrupole ICP-MS, three scan lines covering an area of 13 mm^2 were used. The reference analysis using UCM detected 1047 NPs, while the ICP-MS detected an average (n=2) of 453 NPs. This corresponds to a transport of approximately 43% of NPs.

For the 193-nm laser ablation system coupled to the TOF mass spectrometer, 0.5 mm × 0.5 mm areas were ablated using random square and grid approaches. The random square approach yielded an average (n=2) of 531 NPs, compared to 1252 from the reference UCM, suggesting a transport efficiency (TE) of 42 %. Using the grid approach, 684 NPs were detected by ICP-MS, while UCM yielded 1493 NPs, suggesting a TE of 46%.

As discussed in the main text, the lower TE observed with the 193-nm laser ablation systems compared to the 213-nm and 2940-nm laser ablation systems stems from NP disintegration during ablation and possibly from the lower sensitivity and higher noise floor of the TOF MS. To isolate the influence of the mass spectrometer and different ablation cells, we paired the 193-nm laser with the quadrupole ICP-MS. Signal transients (Figure S7A,B) reveal that the contribution of TOF mass spectrometer to TE reduction was minimal, causing only a slight reduction in signal. This indicates that ablation-related phenomena remain the dominant factor affecting TE for the 193-nm laser system.

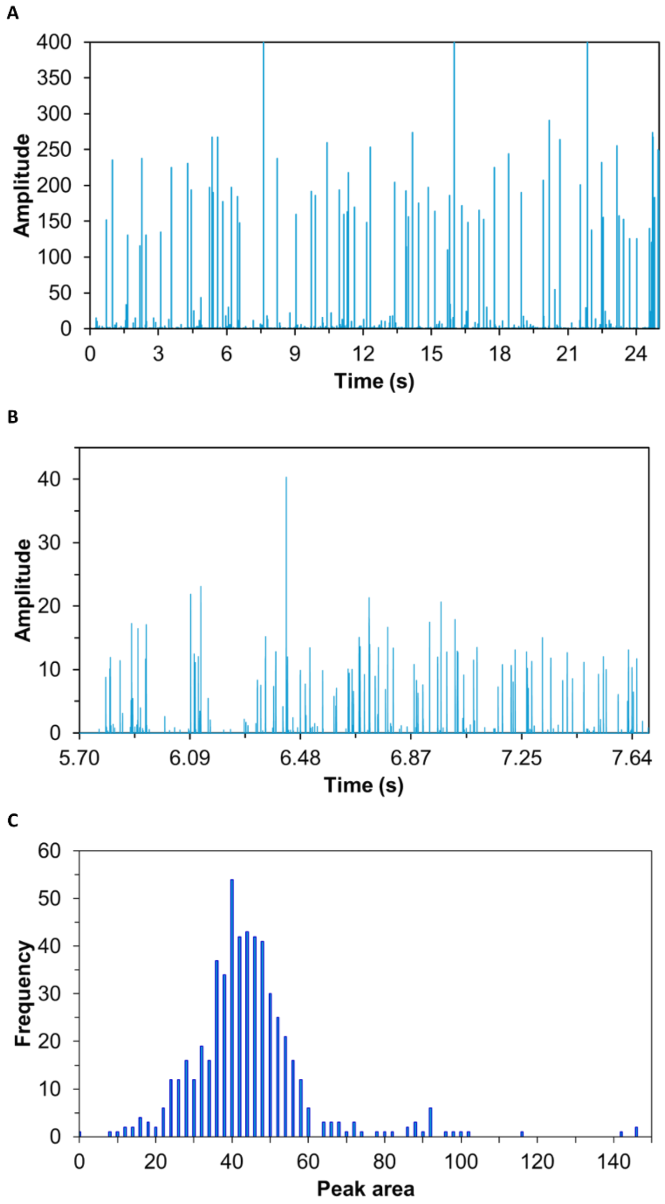

Figure S8. Signal transients from the 193-nm laser ablation system coupled to (A) quadrupole MS and (B) TOFMS. The elevated baseline near NP peaks is slightly lower for the quadrupole signal compared to the TOFMS signal. (C) NP peak area histogram from 193-nm LA-SP-ICP-TOFMS obtained from a 0.5 mm × 0.5 mm ablation area.

References

(1) Homann, C.; Krukewitt, L.; Frenzel, F.; Grauel, B.; Würth, C.; Resch-Genger, U.; Haase, M. *Angew. Chem. Int. Ed.* **2018**, *57*, 8765–8769. https://doi.org/10.1002/anie.201803083

(2) Kraft, M.; Würth, C.; Palo, E.; Soukka, T.; Resch-Genger, U. Methods Appl. Fluoresc. 2019, 7 (2), 024001. https://doi.org/10.1088/2050-6120/ab023b

(3) Hlaváček, A.; Křivánková, J.; Brožková, H.; Weisová, J.; Pizúrová, N.; Foret, F. Anal. Chem. 2022, 94 (41), 14340–14348. https://doi.org/10.1021/acs.analchem.2c02989

(4) Hlaváček, A.; Farka, Z.; Mickert, M. J.; Kostiv, U.; Brandmeier, J. C.; Horák, D.; Skládal, P.; Foret, F.; Gorris, H. H. Nat. Protoc. 2022, 17 (4), 1028–1072. https://doi.org/10.1038/s41596-021-00670-7

(5) Hlaváček, A.; Uhrová, K.; Weisová, J.; Křivánková, J. Anal. Chem. 2023, 95 (33), 12256–12263. https://doi.org/10.1021/acs.analchem.3c01043

(6) Lockwood, T. E.; Gonzalez de Vega, R.; Du, Z.; Schlatt, L.; Xu, X.; Clases, D. J. Anal. At. Spectrom. 2024, 39 (1), 227–234. https://doi.org/10.1039/D3JA00288H

(7) Lockwood, T. E.; Schlatt, L.; Clases, D. J. Anal. At. Spectrom. 2025, 40 (1), 130–136. https://doi.org/10.1039/D4JA00241E
